# Light-induced Conductance Switching in Photomechanically Active Carbon Nanotube-Polymer Composites

**DOI:** 10.1038/s41598-017-10211-6

**Published:** 2017-08-29

**Authors:** V. Schneider, O. Polonskyi, T. Strunskus, M. Elbahri, F. Faupel

**Affiliations:** 1 Chair for Multicomponent Materials, Faculty of Engineering, Kiel University, Kaiserstr. 2, 24143 Kiel, Germany; 2 Nanochemistry and Nanoengineering, Department of Chemistry & Materials Science, Aalto University, Kemistintie 1, C321, 00076 Aalto, Finland

## Abstract

Novel, optically responsive devices with a host of potential applications have been demonstrated by coupling carbon nanomaterials with photochromic molecules. For light-induced conductance switching in particular, we have recently shown that carbon nanotube-polymer nanocomposites containing azobenzene are very attractive and provide stable and non-degradable changes in conductivity over time at standard laboratory conditions. In these composites, the photoswitching mechanisms are based on light-induced changes in electronic properties and related to the Pool-Frenkel conduction mechanism. However, no link between conductivity switching and the molecular motion of azobenzene chromophores could be found due to application of high elastic modulus polymer matrices. Here we report on single wall carbon nanotube-polymer nanocomposites with a soft polycaprolactone polymer host. Such a system clearly shows the transfer of light-induced, nano-sized molecular motion to macroscopic thickness changes of the composite matrix. We demonstrate that these photomechanical effects can indeed overshadow the electronic effects in conductivity switching behavior and lead to a reversion of the conductivity switching direction near the percolation threshold.

## Introduction

Over the last few decades, many functional small molecules have been developed which have paved the way to a new class of stimuli-sensitive materials. Especially triggering by light seems to be of particular interest due to the ability of wireless control, a broad variety of available functional molecules^1^, and the capability of relatively facile control of wavelength, duration, and localization. Here, typically different wavelengths are used to reversibly change between different isomer states of the smart molecules. Therefore it is not surprising that photoswitchable molecules have been intensively investigated in fields ranging from photomechanics to adhesion to plasmonics and electronics^2–6^. Regarding light-induced conductivity switching, intense research has mainly involved two chromophore types. Azobenzenes, on the one hand, allow reversible control of molecular structure and dipole moment^7^. In diarylethenes, on the other hand, these property changes are less pronounced, and instead they exhibit a strong change in intrinsic conductivity between both isomers.

Different approaches to investigating conductivity switching can be found in the literature. Several reports have described molecular junctions that include diarylethene and azobenzene molecules^8–12^. Overall, very high switching amplitude ratios were achieved. For instance, numbers ranging from 6 to 7000 were reported. However, the feasibility of producing single molecule or monolayer structures on an industrial scale is questionable, at least in the near future.

In contrast, combining photochromic molecules with carbon nanomaterials appears more promising^13^. Quite a few publications deal with field effect transistors on the basis of carbon nanotubes (CNTs) where the change in electrical dipole moment of the two reversible chromophore states is used to modify the electrostatic environment of the tubes, but comparably lower switching effects with unstable amplitudes over time were achieved^14–16^. Another approach is based on carbon nanotube/chromophore hybrid materials^17, 18^. While in an azobenzene hybrid the switching effects were explained in terms of geometrical rearrangement and variation of tunneling barrier between tubes^17^, in a spironaphthoxazine system resistance changes were reported as a result of chromophore HOMO-LUMO level manipulation in combination with a charge transfer mechanism^18^.

An interesting approach was presented by the group of Ben Feringa where a 2D gold nanoparticle network was bridged with intrinsically conductive diarylethene derivatives^19^. More recently, Sciascia *et al*. presented a diarylethene polymer composite where single-wall carbon nanotubes (SWCNT) were intercalated^20^. Both approaches used the intrinsic conductivity changes of the two isomers states. However, one drawback in diarylethene systems is the reported photodecomposition under excessive UV irradiation^19, 21^. In addition, the unequal conversion ratio between the two isomer states decreases the switching amplitude due to enrichment of one photostationary state^19^. These difficulties are of course not a principle limitation and could be solved by chemists in the future. Nevertheless, they motivated us to develop systems that are based on azobenzene derivatives and use simple and reliable preparation techniques which are easily scalable to industrial processing. Recently, we reported on metal-polymer nanocomposites where azobenzene derivatives were used to photoswitch the spacing between metal nanoparticles and thus the tunneling conductance, which depends exponentially on the tunneling distance^22^. The change in free volume upon photoswitching was demonstrated by positron annihilation lifetime spectroscopy^23^. Although a constant switching amplitude was achieved, the switching ratio did not exceed one percent. By extending our approach to carbon nanotube-polymer composites, the switching amplitude could be increased by a factor of about 30 near the percolation threshold with good device stability in amplitude and performance over time^24^. However, the switching mechanism now proved to be electronic in nature and based on a Pool-Frenkel type conduction, where the chromophores are an essential part of the conduction pathway. We also investigated multi-wall carbon nanotube-polymer composites, using the same azobenzene derivative, which showed the opposite switching direction above the percolation threshold and weaker overall performance as their SWCNT counterparts^25^. Again, the photoswitching mechanism was attributed to light-induced changes in electronic properties.

The abovementioned investigated systems of polymer composites with CNTs showed that the electronic effects dominate for photoswitching of conductivity and there was no evidence for participation of a geometrical effect. On the over hand, the results of Mativetsky *et al*.^9^ obtained for metal-molecule-metal junctions with azobenzenes suggest that large switching ratios could be achieved if one were able to transfer the geometrical effect to CNT polymer composite systems, i.e. if one could achieve significant changes in the tunneling gap separation in composites close to the percolation threshold. Whether this approach will be successful is a very interesting question, since the geometrical effect must compete with the other abovementioned mechanisms of light-induced conductance switching found in CNT-based systems so far.

Here we show, using single wall carbon nanotube-polymer nanocomposites with a soft polycaprolactone matrix, that photomechanical effects can indeed dominate the conductance switching behavior near the percolation threshold. Moreover, we demonstrate a resulting reversion of the switching direction at higher SWCNT concentration. The results are discussed in terms of competing geometrical and electronic effects. Polycaprolactone (PCL) was chosen as a host matrix because it has a low bulk elastic modulus of 300 to 500 MPa^26, 27^, it is soluble in common organic CNT solvents, and it is miscible with the present azobenzene derivative and does not show phase separation.

## Results and Discussion

To obtain a photomechanical transfer of molecular azobenzene to the polymer matrix, some requirements should be met. First, the mechanical properties of the polymer matrix are very important. Hügel *et al*. and Holland *et al*. showed in single molecule force spectroscopy experiments that isomerization of azobenzene takes place only under finite external force^28, 29^. However, in stiff polymer matrices, isomerization reactions occur regardless of mechanical constraints due to free volume present in polymer films^23, 30, 31^. Harms *et al*. could verify that isomerization of azobenzene is accompanied by free volume changes in PMMA on the order of 10%^23^. But such steric constraints strongly influence reaction kinetics so that isomerization of azobenzene typically proceeds several orders of magnitude slower than in solutions^32–37^. On the other hand, a proper coupling between azobenzene and the matrix is needed to transfer the molecular motion. Such coupling can be achieved by a chemical bond or by using alkenes as bulky side branches grafted to the azobenzene, as in our case. In our previous experiments, high elastic modulus polymers like poly(methylmethacrylate) (PMMA) or polystyrene were used^22, 24, 25, 35^. In such systems rather small or localized transfer of molecular motion was achieved^22, 36^. Therefore, a change to polymer matrices with reduced elastic modulus, like polycaprolactone (PCL),was inevitable.

**Figure 1:**
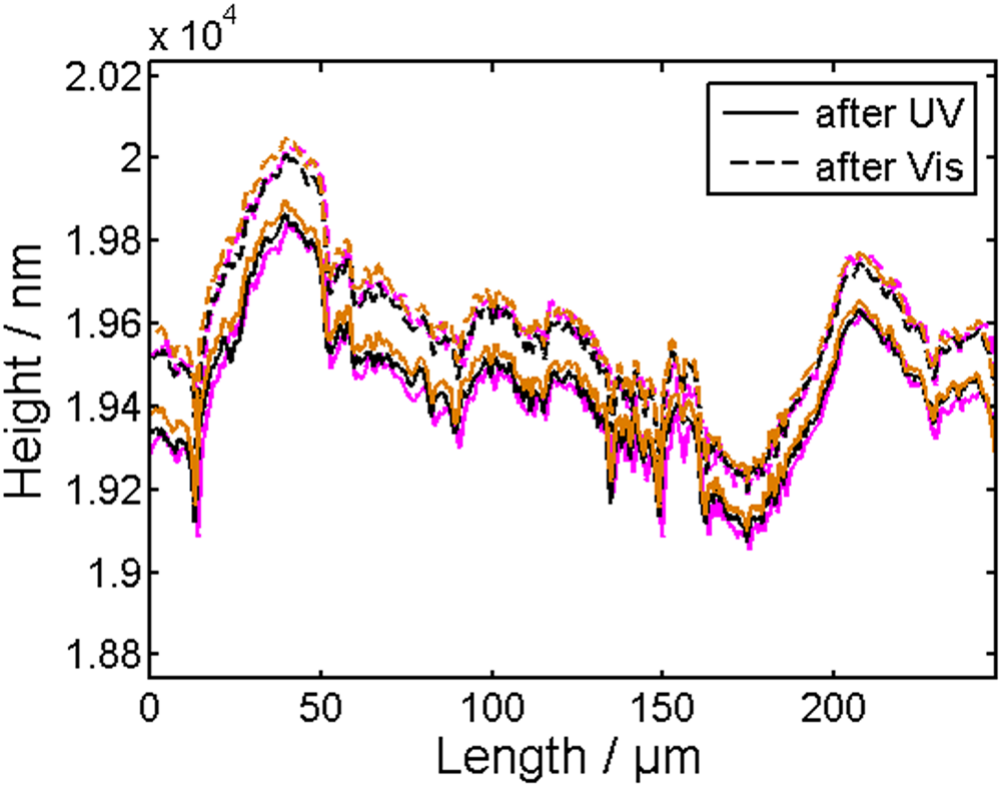
PCL/Azobenzene composite film measured with a profilometer after consecutive illumination with UV and Vis light. The solid lines correspond to profiles measured after UV and dashed lines to profiles measured after Vis illumination. Different colors represent different successive UV/Vis cycles.

Initially, the shrinkage/expansion performance of the chromophore-polymer blends was investigated in response to the isomerization reaction without SWCNTs. For this, a drop-casted PCL/azobenzene composite film with 20 wt% chromophore was prepared on a glass substrate. Here, we used a rather thick film in comparison to the samples employed for the conductivity measurements discussed below, because it was necessary to make the expected small dimensional changes visible in a 2D profile of the profilometer within its resolution limit. Using a 2D profile provides additional information and provides the capability to check whether the effect is evenly distributed over the surface. Especially the performance at asperities can give hints to bulk or surface effects. The measured average layer thickness of the film amounted to 19.42 ± 0.176 µm, as shown in Fig. 1. Successive illumination for 5 min with unpolarized UV and visible light produced a reversible response in composite film thicknesses of 167 ± 35 nm, on average, which corresponds to a thickness change of 0.85%. Every thickness measurement was conducted after a corresponding illumination cycle, while the sample was left in the dark. It is important to mention that the film thickness shrinks under UV-light and increases under visible. Such behavior is expected because of the shortening of the distance between both benzene rings in the molecule after UV illumination (see Supplementary Information Figure S6)^38^. The measured variation in thickness is a reversible and nonlocal microscopic effect in contrast to our previous report^36^. The 2D-profile is very beneficial and shows prominent changes over surface length as much as several hundred micrometers, which is absent in PCL matrix without azobenzene derivatives. One observes that all asperities are almost equally lifted, while a pronounced broadening of asperities is not visible. Such an observation suggests a bulk effect is responsible for the described changes, i.e. an accumulation of small molecular motion transfer over the film thickness.

**Figure 2:**
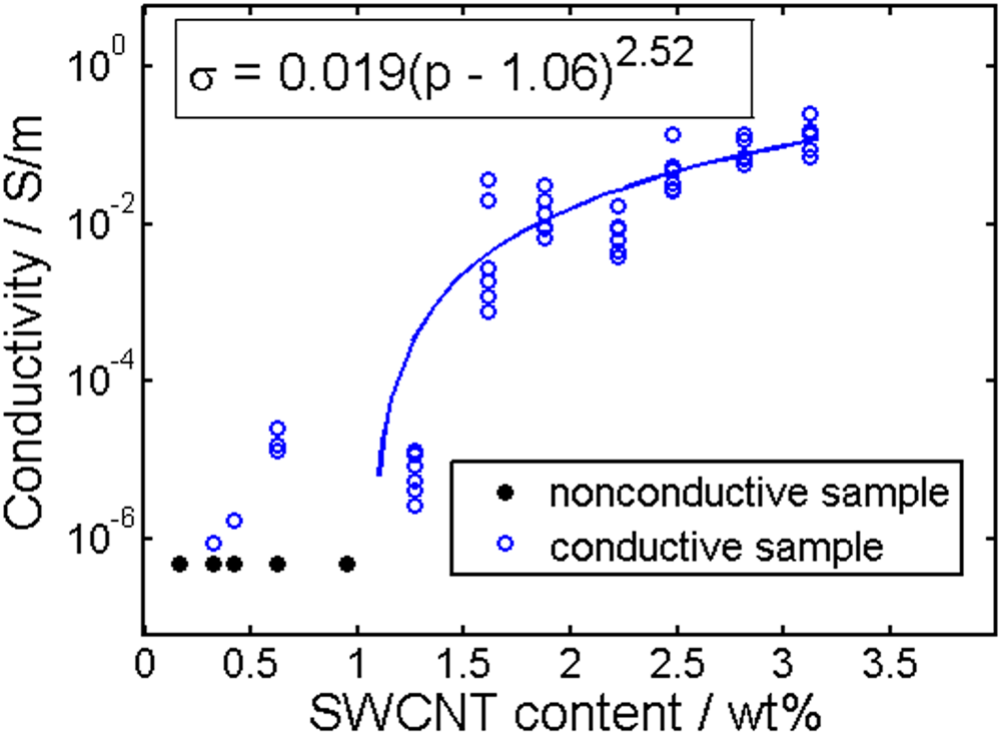
Percolation curve for twelve batches of PCL/Azo/SWCNT composites. The solid line is a fit to the equation shown in the inset, which follows from percolation theory. The resulting prefactor and exponent are 0.019 ± 0.045 S/m and 2.52 ± 1.4, respectively. The error given corresponds to a confidence interval of 68%.

Having proven that the photomechanic conversion in the chromophore blended polymer matrix works, we next studied the influence of molecular motion on conducting properties. Therefore, twelve batches with PCL as the polymer matrix, 20 wt% of azobenzene derivative and consecutively increasing amounts of SWCNT’s (0.16–3.12 wt%) were prepared. A detailed composition can be found in the Supplementary Information. The films were cast by spin coating and resulted in 400 nm thick films. This film thickness was chosen to provide transparent films even at the highest SWCNT loadings for the illumination cycles. Figure 2 displays the measured conductivities as a function of the SWCNT content. One clearly notes a transition from nonconductive to conductive composites. Solid black circles symbolize nonconductive samples whose conductivity was below the measurement range of our setup, while outlined blue circles stand for conductive specimens. The solid blue line represents a fit to conductive samples in accordance with the formula σ ~ (*p*-*p*_c_)^*t*^ from percolation theory^39, 40^, where *p*, *p*_c_, and *t* are SWCNT content, percolation threshold, and critical exponent, respectively. Corresponding fit values are displayed in the figure. According to the fit, the percolation threshold is at 1.06 ± 0.83 wt% SWCNT concentration and the critical exponent is 2.52 ± 1.4, which are reasonable values for such composites^39^. The error given corresponds to the confidence interval of 68%.

**Figure 3:**
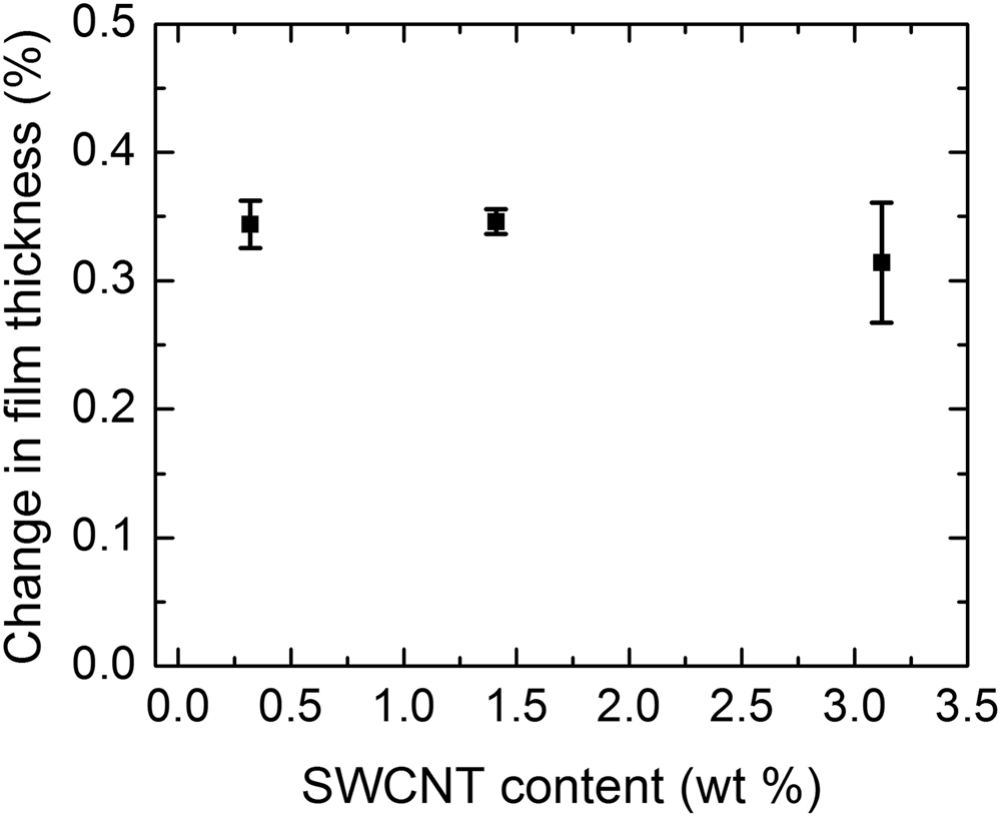
Relative change in composite thickness with SWCNT content due to UV/Vis illumination cycles. Shrinkage occurred after UV illumination and expansion after visible light illumination. Error bars represent standard deviations.

To confirm that the light-induced molecular motion transfer to the polymer matrix still works in the SWCNT containing composite as in the PCL/Azobenzene device, three characteristic devices with 0.32, 1.3, and 3.2 wt% SWCNT content (i.e., below, above and in the transition region of the percolation curve) were tested by angle-resolved ellipsometry measurements. Ellipsometry was chosen because profilometry did not provide sufficient resolution for 400 nm thin SWCNT composite films. As shown in Fig. 3, the relative change in film thickness is between 0.3–0.35% for all SWCNT batches, while no changes were found in pure PCL matrix. Such light-induced film thickness variation in contrast to the 0.8% in the PCL/Azobenzene device without SWCNTs is not unexpected. The reduction may originate from the increased substrate clamping in the much thinner polymer film and the well-known reinforcement effect of the SWCNT filler. On the other hand, the photomechanic behavior of the SWCNT composites correlates well with the performance shown in Fig. 1, namely the contraction of composite thickness under UV and expansion under Vis illumination. Interestingly, the SWCNT reinforcement does not seem to cause a large concentration dependence of the photomechanics in the investigated SWCNT concentration range. Figure 3 only suggests a minor decrease for the specimen with the highest content. Such behavior is probably due to the extremely high aspect ratio of the SWCNTs and the quasi-2D character of the composite which gives rise to an in-plain alignment.

**Figure 4:**
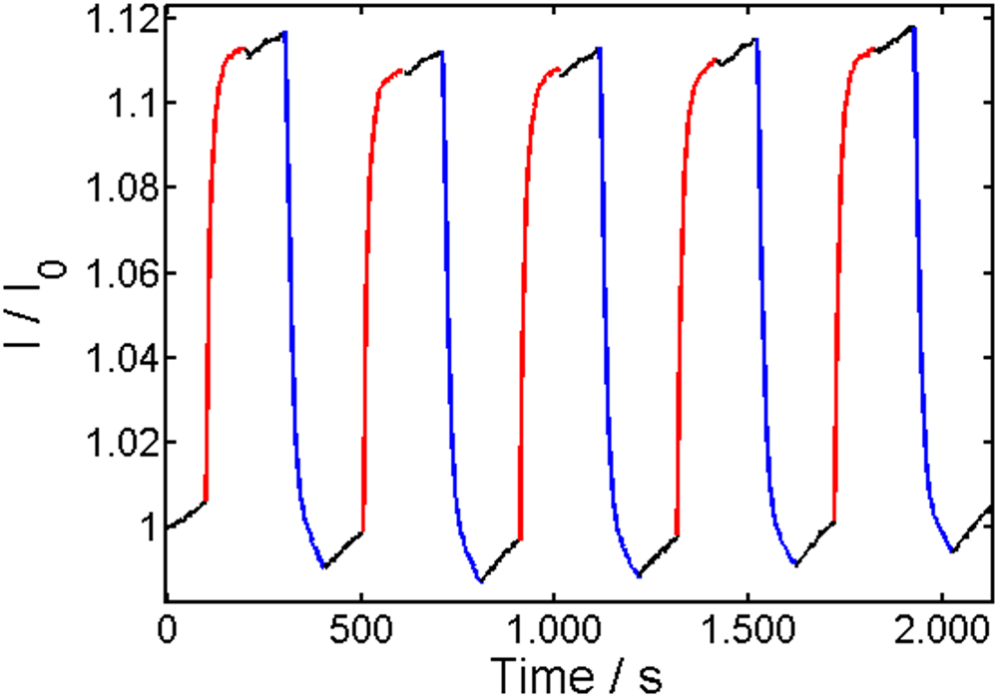
Typical response to UV/Vis illumination of samples in the top right corner of the percolation curve. Here a sample with 2.8 wt% SWCNT is shown, for instance. Red color stands for illumination time period with 475 nm, blue for 375 nm, and black for no illumination. *I*_0_ equals 4.15 × 10^−7^ A.

**Figure 5:**
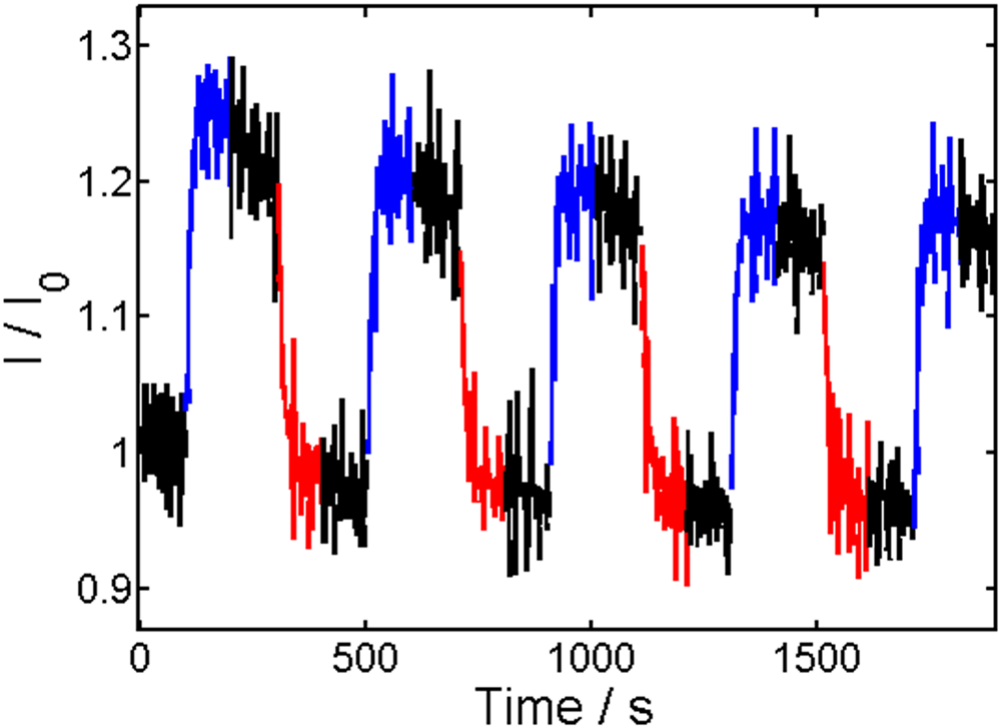
Typical response to UV/Vis illumination of samples in the lower left corner of the percolation curve. Here, a sample with 1.3 wt% is shown for example. Red color stands for illumination time period with 475 nm, blue for 375 nm, and black for no illumination. *I*_0_ equals 2.96 × 10^−10^A.

**Figure 6:**
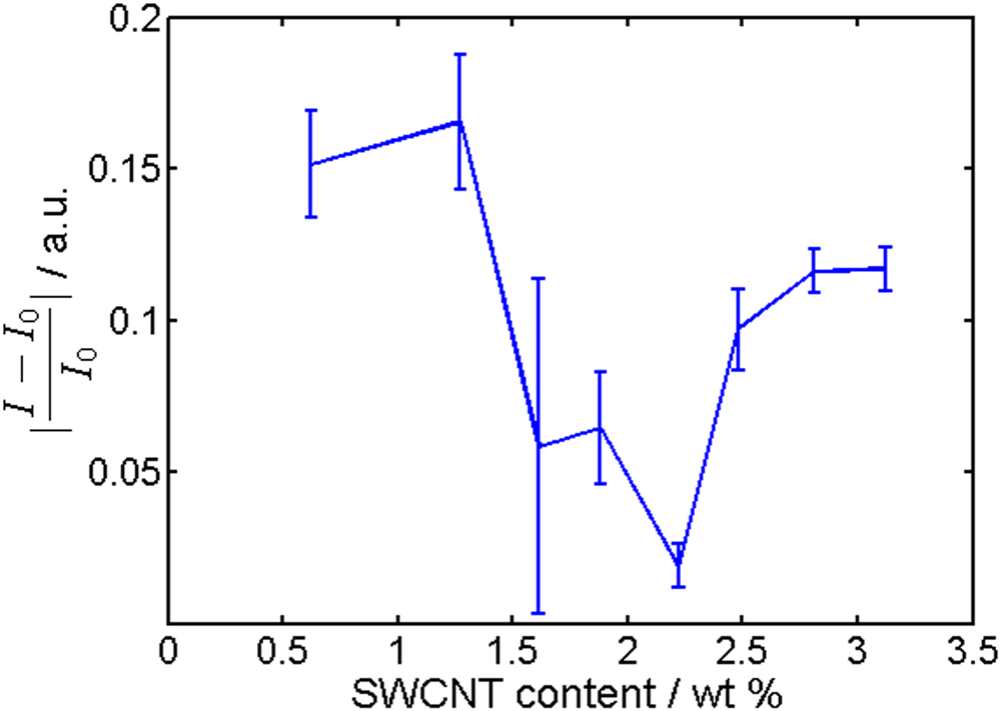
Measured relative switching amplitudes of current between UV/Vis illumination cycles. Errorbars represent standard deviations. A pronounced decrease in current switching amplitude is observed near the percolation threshold.

In the next step, the electro-optical properties of the conductive samples were investigated. For this, specimens were consecutively illuminated 100 seconds with 375 nm, held 100 seconds in the dark and illuminated 100 seconds with 475 nm for multiple times while the current was continuously measured. We mention that two of the conductive samples with the lowest SWCNT concentration showed no switching at all. The response in conductivity of the other specimens was characterized by two opposite switching directions, as can be seen in Figs 4 and 5, for instance. In the top right corner of the percolation curve (see Fig. 2), we found a decrease in conductivity with UV light illumination at 375 nm and an increase with visible illumination at 475 nm. This is illustrated in Fig. 4. Approaching the percolation threshold, we saw a deviation from this behavior. Starting at approximately 2.2 wt% SWCNT content, a mixed behavior among different samples was found. Particularly, a reversed response with an increase in conductivity under illumination with 375 nm and a decrease under illumination with 475 nm was seen in addition to the behavior shown in Fig. 4. This behavior was accompanied with overall lower conductivity changes under illumination. While a mixed response was found in the transition region, at the bottom left corner of the percolation curve (see Fig. 2) only the opposite switching direction to Fig. 4 appeared, as illustrated in Fig. 5, for instance. An overview of the corresponding relative switching amplitudes, shown in Fig. 6, gives more insight into the switching behavior. Here, the absolute value of the relative switching amplitude is displayed. The absolute value was chosen for clarity, since otherwise mixed positive and negative data points would have occurred in the transition region. One notes a decrease of the absolute switching amplitude while approaching the percolation threshold and an increase again after the transition. The minimum of the relative switching amplitude is to be expected for two competing switching mechanisms with opposed direction in conductivity changes above and below the percolation threshold.

To gain more insight into the underlying switching mechanisms, we investigated the *I*(*V*) characteristics of specimens. All conductive samples showed strong nonlinear behavior in the investigated voltage range, which can originate from different conduction mechanisms. Reported mechanisms for CNT composites in the literature are Motts variable range hopping (VRH), Sheng’s fluctuation induced tunneling (FIT), and Pool-Frenkel (PF) or space charge limited conduction (SCLC), for instance^41–45^.

**Figure 7:**
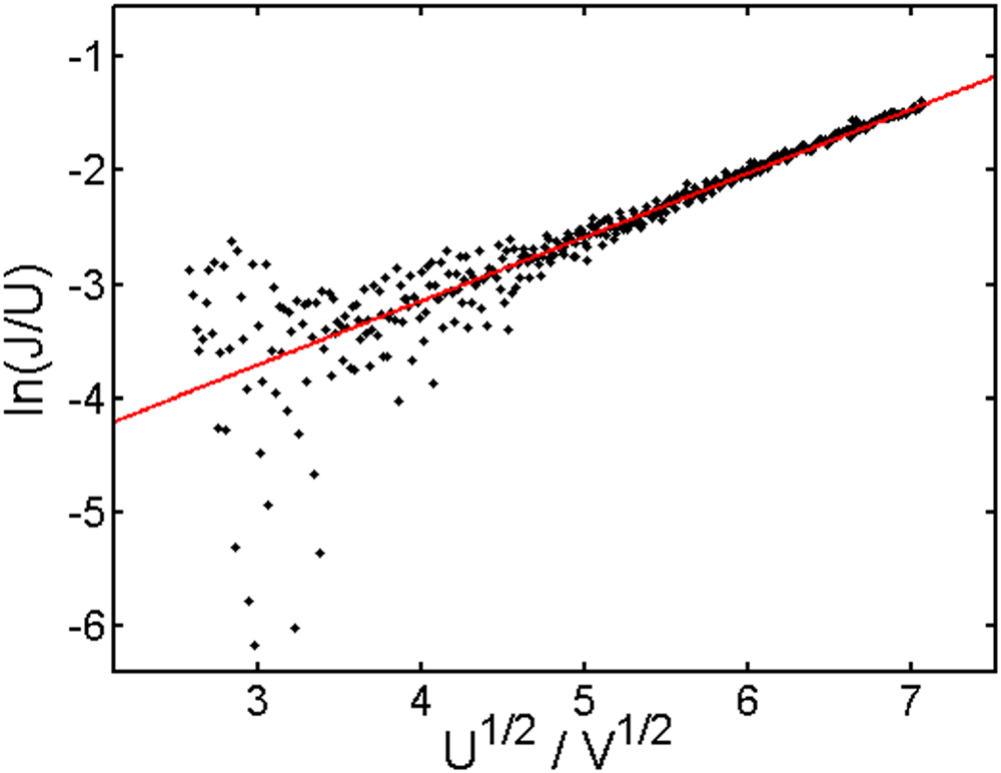
Pool-Frenkel plot for devices with 1.3 wt% SWCNT content. The red line corresponds to a linear fit resulting in a *y*-intercept of −5.4 ± 0.067 and a slope of 0.56 ± 0.013. The error given corresponds to a confidence interval of 68%.

For specimens below 1.5 wt% SWCNT content, a Pool-Frenkel dependence was found, which is characterized by^43^:

$$J\propto U\exp (\beta {U}^{\frac{1}{2}})$$

where *J* is the current density, *U* the corresponding voltage, and *β* the barrier lowering coefficient. A Pool-Frenkel dependence is typically confirmed by plotting ln(*J*/*U*) vs. *U* ^1/2^, where beta is extracted as the slope of the curve. The corresponding plot is shown in Fig. 7. The extracted linear fit values are a ***y***-intercept of −5.4 ± 0.067 and a slope of 0.56 ± 0.013. The given errors correspond to confidence intervals of 68%.

**Figure 8:**
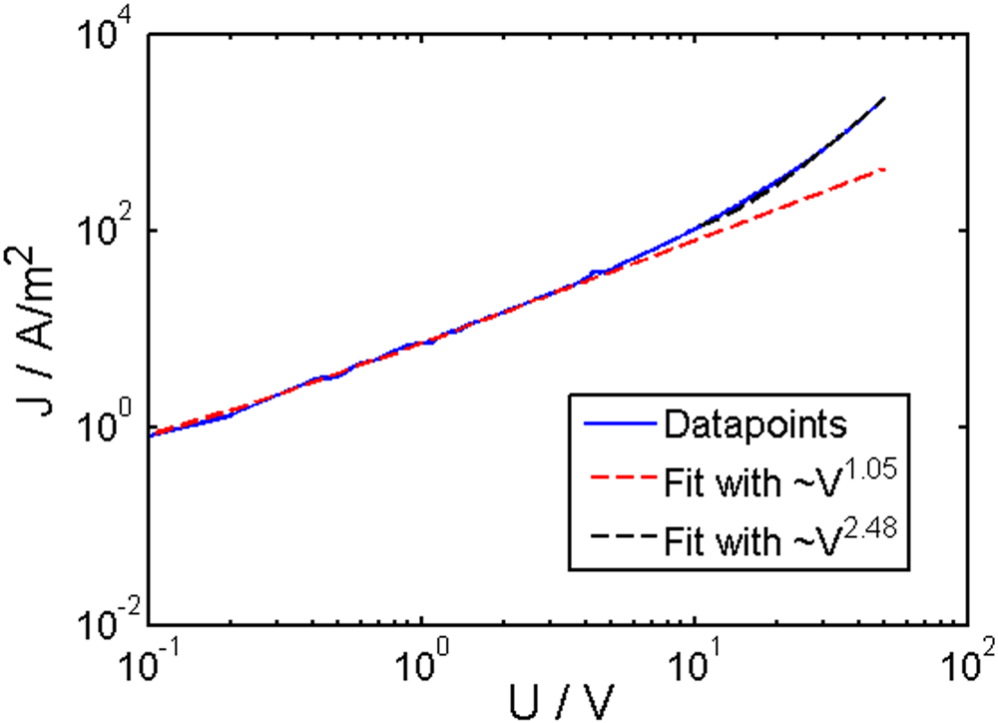
Typical log-log plot of the *J*(*V*) relation for specimens with a SWCNT content above 1.5 wt%. The dashed lines correspond to fit functions with power law dependance. The resulting exponents are 1.05 ± 0.048 in the low voltage regime and 2.48 ± 0.008 at higher voltage. The given error corresponds to a confidence interval of 68%.

For more conductive specimens with higher SWCNT content, the conduction mechanism deviates, as shown on Fig. 8. On a double logarithmic plot, the most probable model is space charge limited conduction. SCLC is characterized by a power law dependency:

$$J\propto a\cdot {U}^{m}$$

where *J* is the current density, α is a constant, *U* the corresponding voltage and *m* the exponent, which can be extracted as the slope of a log-log plot of *J* vs. *V*. In general, devices show an ohmic region with increasing voltage as long as the material can supply enough free charge carriers for conduction, as appears to be the case at lower voltages in our devices as shown in Fig. 8. The characteristic exponent from a power law fit in this area is 1.05, which clearly indicates ohmic behavior. In the region between 3 V to 6 V, a transition takes place and the power law exponent increases to a value of 2.48. This transition is expected if the composite can no longer provide enough free charge carriers, and the current becomes dominated by charge carriers injected from the contact electrodes. Since the charge mobility in the composite is much lower than in the metal electrodes, charge accumulation occurs at the metal/composite interface, which leads to the observed power law dependence. The exact nature of the SCLC mechanism is strongly related to the type of traps, their distribution, and position with respect to the Fermi level, which is generally difficult to predict. However, for shallow traps an exponent of 2 is typical (Mott-Gurney law) and higher numbers are expected for the trap-filled regime. Hence the exponent obtained in our composites seems to be very reasonable.

The present findings on the conduction mechanism correlate well with our previously reported results on PMMA/azobenzene/SWCNT composites^24^, where the azobenzene units were identified as an integral part of the conduction paths. The Pool-Frenkel conduction mechanism seems to dominate when the conducting network is loosely connected. Therefore, the Pool-Frenkel behavior at the percolation threshold is not surprising. At higher SWCNT concentration well beyond the percolation threshold, a change to ohmic behavior occurs due to better electrical connectivity and the buildup of percolative conduction paths. However, with increasing voltage at a particular current density, charge accumulation occurs due to the low charge carrier mobility in the composite, and leads to space charge limited conduction.

Since conduction mechanism is comparable to our previous findings^24^ similar performance in photoswitching is not surprising. Great similarity in regard to conductivity changes, and switching direction under consecutive UV/Vis illumination cycles show specimens with higher content of SWCNTs (in the top right corner of the percolation curve in Fig. 2, in particular). However, the change in the conductance switching direction near the percolation threshold (compare Figs 4 and 5) and the reduction of the relative switching amplitude (Fig. 6) at the percolation threshold are clearly related to the photomechanic expansion/contraction of the composite film, since this is the new feature incorporated by soft polymer matrix in comparison to our previous report^24^. The observed photocontraction of the composites’ thickness under UV illumination goes hand in hand with the resulting increase in conductivity for specimens below the percolation threshold as shown in Fig. 5 and hence immediately explains the inversion of the conductance switching direction compared to samples, as shown in Fig. 4.

In these terms, the behavior in Fig. 6 can be explained in the following way. While in the top right corner of the percolation curve the mechanism is dominated by electronic effects as discussed in our previous work^24^, a reduction in the SWCNT content leads to a decreased number of parallel conduction paths. It seems that this reduction is needed for the photomechanic effect to become the dominant mechanism. In contrast, the present new feature of photo expansion/contraction of the soft polymer matrix is more or less independent of the SWCNT content in the relevant range (see Fig. 3). When the conductive network is diluted to such an extent that conduction through the polymer via a Pool-Frenkel mechanism becomes the current limiting mechanism, the influence of photoexpansion/photocontraction on charge transfer characteristic seems to take over the dominant role.

## Conclusion

We extended our previous work on light-induced conductance switching of azobenzene-containing, near-percolated single wall carbon nanotube-polymer composites to composites with a soft, low *T*_g_ thermoplastic polycaprolactone matrix that showed pronounced intrinsic photomechanic activity. In particular, light-induced molecular motion of azobenzene chromophores could be successfully coupled to reversible macroscopic thickness changes on the order of 0.85% for PCL/azobenzene and 0.3–0.35% for SWCNT-containing polymer composites. The photomechanic effect clearly turned out to affect the photoswitching behavior of the conductance in the region of the percolation threshold. Here, a reversion in the light-induced conductance switching direction with UV and visible light illumination was detected. The origin of this transition is explained in terms of competing photomechanic and electronic effects. Moreover, below the percolation threshold the geometric effect indeed takes the dominant role in conductivity switching. Stable operation over time at standard laboratory conditions without degradation of the conductivity switching amplitude are further advantages of the present system.

## Methods

### Materials

The carboxyl-group-functionalized single-wall carbon nanotubes (product number: 652490–250MG) and the polycaprolactone polymer (product number: 440744–5 G) were purchased from Sigma Aldrich. The SWCNTs had a mean diameter of 1.5 nm and a mean length of 2 µm, respectively. The polycaprolactone (PCL) polymer pellets had a number average molecular weight of 70–90 kg/mol and a density of 1.145 g/mol. The solvent dimethylformamide was purchased from Carl Roth GmbH (ROTIPURAN 99.8%). The synthesis and the optical properties of the azobenzene chromophores (4-hexyl-phenyl-[4-(propyl-butoxy)-phenyl]-diazene) are described elsewhere^35^.

### Preparation of specimens

For composite preparation, first 1 g of PCL polymer was dissolved in 20 ml DMF resulting in a concentration of 50 mg/ml. It is important to note that for complete dissolution the liquid was held at about 50° Celsius and magnetically stirred for 1 h until the solution became clear. A Whatman PTFE filter with 0.45 µm pore size was used to extract remaining undissolved polymer particles. Cooling of the liquid leads to slow agglomeration of polymer chains resulting in a milky solution. To avoid agglomeration, the azobenzene chromophore was then added to the solution without cooling until it accounted for 20 wt% of the polymer-chromophore base. Further 1 ml of that solution was added to preweighed batches of SWCNT, where the carbon nanotube content was varied between 0.16 and 3.1 wt% with respect to the whole weight of the polymer, chromophore, and SWCNTs. The exact values of constituents in solutions can be found in the Supplementary Information. The dispersion of the SWCNT was achieved by a consecutive treatment with a 500 W tip sonicator from Sonorex at 20% amplitude for 1.5 min (6 mm tip was used) plus an ultrasound bath for 50 min which was held at 50 °C during sonication. Spin coating was performed directly afterwards.

### Characterization

As substrates, 1 cm² soda lime glass was used. For electrical characterization, Cr/Au (10 nm/ 100 nm) contact bars, 500 µm long, 50 µm wide and with a separation 50 µm were fabricated with standard UV-lithography. These dimensions of the contact pads were selected in accordance to the SWCNT size and electrical resolution of our equipment. Prior to spin coating, the substrates were cleaned in an ultrasonic bath for 5 min first with acetone and then DI-water. Afterwards cleaning in RCA-1 (80 °C, 40 min) and DI-water for 5 min in an ultrasonic bath took place. Spin coating was always performed under a nitrogen atmosphere to ensure similar atmospheric lab conditions.

A profilometer from Bruker, Model Dektak XT,with a stylus radius of 2 µm was used for thickness measurements of the PCL/Azobenzene composite. The electro optical characterization setup consisted of a 500 W Xe-lamp, a monochromator from Newport (model number: 74100), and a Keithley 6487 picoamperemeter. The intensities used were 2.66 mW/cm² at 475 nm (Vis) and 2.63 mW/cm² at 375 nm (UV). The leakage currents in the electrical setup were as low as 5 × 10^−11^ A. For photomechanic expansion experiments, two LABINO handheld lamps were used as light source (MPXL-UV-H135 and MPXL-White-H135). Variable angle spectroscopic ellipsometry reflection measurements were performed on a J.A. Woollam Co., Inc. M2000 UI to track photomechanic expansion in composite samples containing SWCNTs. The typical thickness of spin-coated specimens containing SWCNTs was around 400 ± 10 nm. Fitting to the ellipsometry data was carried out in 700 nm to 1600 nm wavelength range.

All data generated in or analyzed during this study are included in this published article and its Supplementary Information files.

## Electronic supplementary material

Supplementary information
