# Supplementary material for: Light-induced Conductance Switching in Photomechanically Active Carbon Nanotube-Polymer Composites
Source: Sci Rep. 2017 Aug 29;7:9648. doi: 10.1038/s41598-017-10211-6 (PMC5575261; doi:10.1038/s41598-017-10211-6)
Supplement: Supplementary file 1 — Supplementary information [file 41598_2017_10211_MOESM1_ESM.pdf]

Light-Induced Conductance Switching in Photomechanically Active Carbon Nanotube-Polymer Composites

V. Schneider,[1] O. Polonskyi,[1] T. Strunskus,[1] M. Elbahri,[2] F. Faupel[1*]

[1]Chair for Multicomponent Materials, Faculty of Engineering, Kiel University, Kaiserstr. 2, 24143 Kiel, Germany

[2]Nanochemistry and Nanoengineering, Department of Chemistry & Materials Science, Aalto University, Kemistintie 1, C321, 00076 Aalto, Finland

Correspondence and requests for materials should be addressed to F.F. (E-Mail: ff@tf.uni-kiel.de)

Supplementary Information

Table S1:The exact values of constituents in twelve solutions used in this paper.

Solvent ml	Polymer mg	SWCNT mg	Azo mg
1.000	50.000	0.100	12.270
1.000	50.000	0.200	12.270
1.000	50.000	0.260	12.270
1.000	50.000	0.390	12.270
1.000	50.000	0.600	12.270
1.000	50.000	0.800	12.270
1.000	50.000	1.020	12.510
1.000	50.000	1.200	12.510
1.000	50.000	1.420	12.510
1.000	50.000	1.590	12.510
1.000	50.000	1.810	12.510
1.000	50.000	2.010	12.510

Polymer wt%	SWCNT wt%	Azo wt%
0.80167	0.00160	0.19673
0.80038	0.00320	0.19641
0.79962	0.00416	0.19623
0.79796	0.00622	0.19582
0.79529	0.00954	0.19516
0.79277	0.01268	0.19455
0.78703	0.01606	0.19691
0.78481	0.01884	0.19636
0.78211	0.02221	0.19568
0.78003	0.02480	0.19516
0.77736	0.02814	0.19450
0.77495	0.03115	0.19389
Standard deviation		
0.009611		0.000968
Meanvalue		
0.78950		0.19562

Top view SEM images taken with a Zeiss Supra 55VP microscope show the good dispersion of different fractions of SWCNT constituent in the polymer composites. For SEM analysis, the batches were consecutively spin coated together with the other specimens on silicon substrates to avoid unwanted charging of the samples. Figs. 9 to 11 show typical examples. For very low SWCNT concentrations below 0.62 wt%, no images could be taken, but dispersion problems are not expected at these low concentrations, anyway.

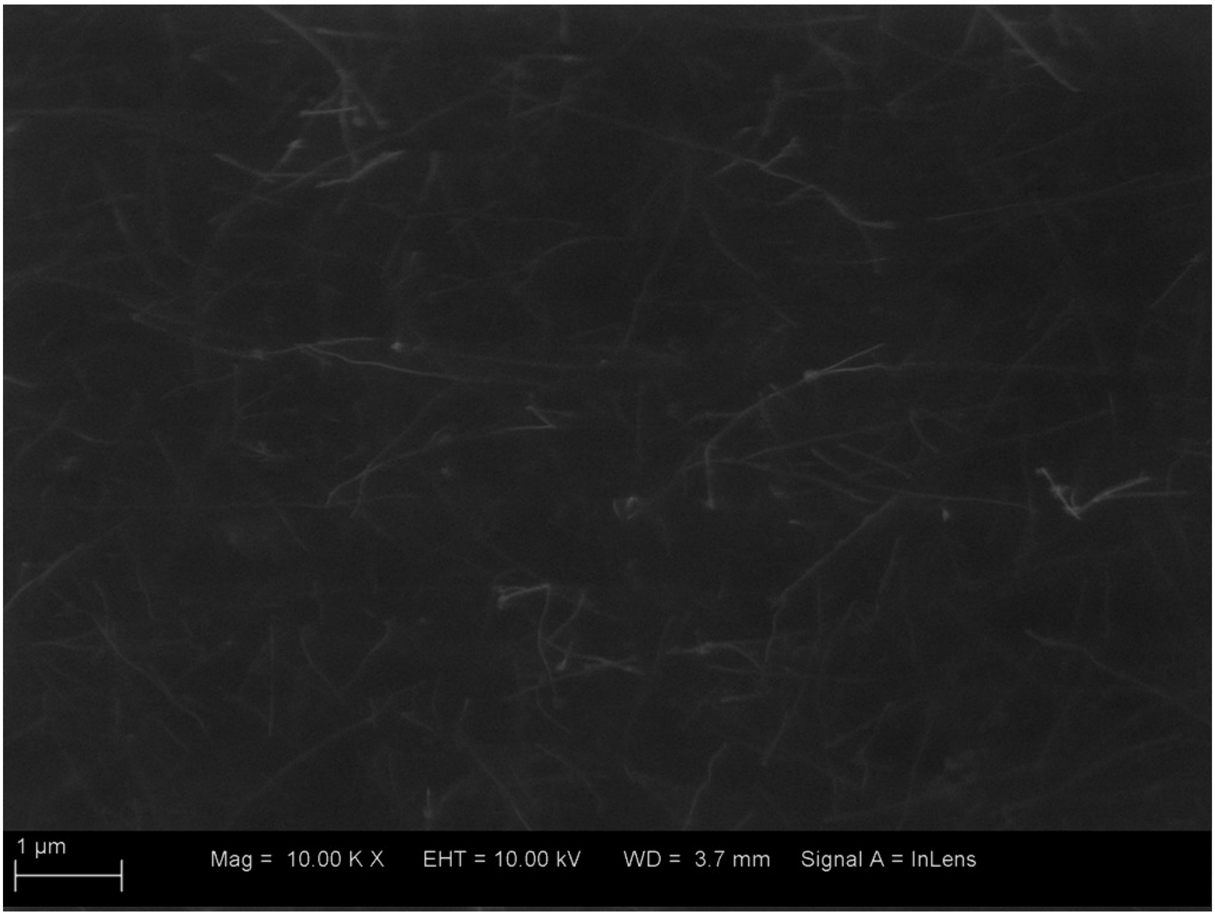

Figure S1: SEM micrograph of a composite with 0.62 wt% SWCNT.

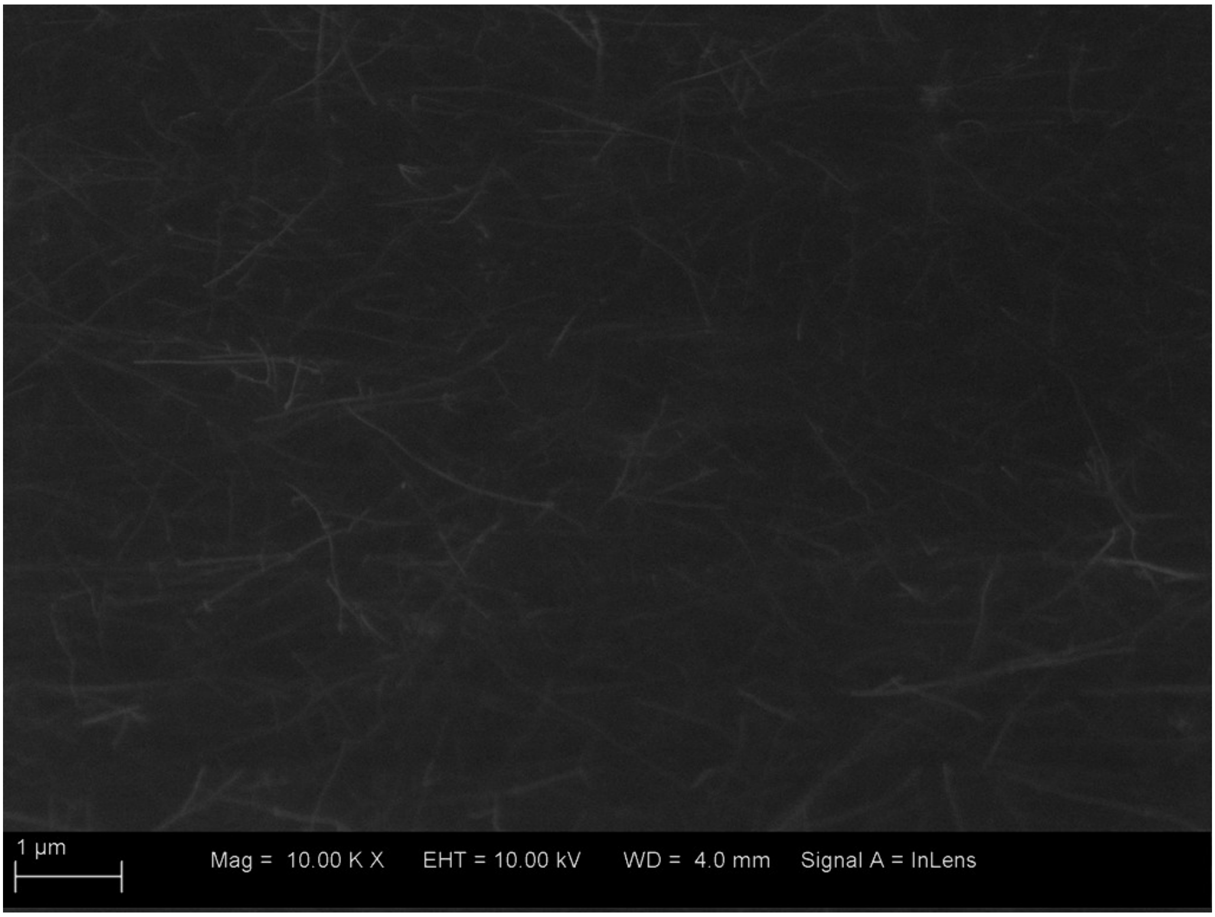

Figure S2: SEM picture of a composite with 0.95 wt% SWCNT.

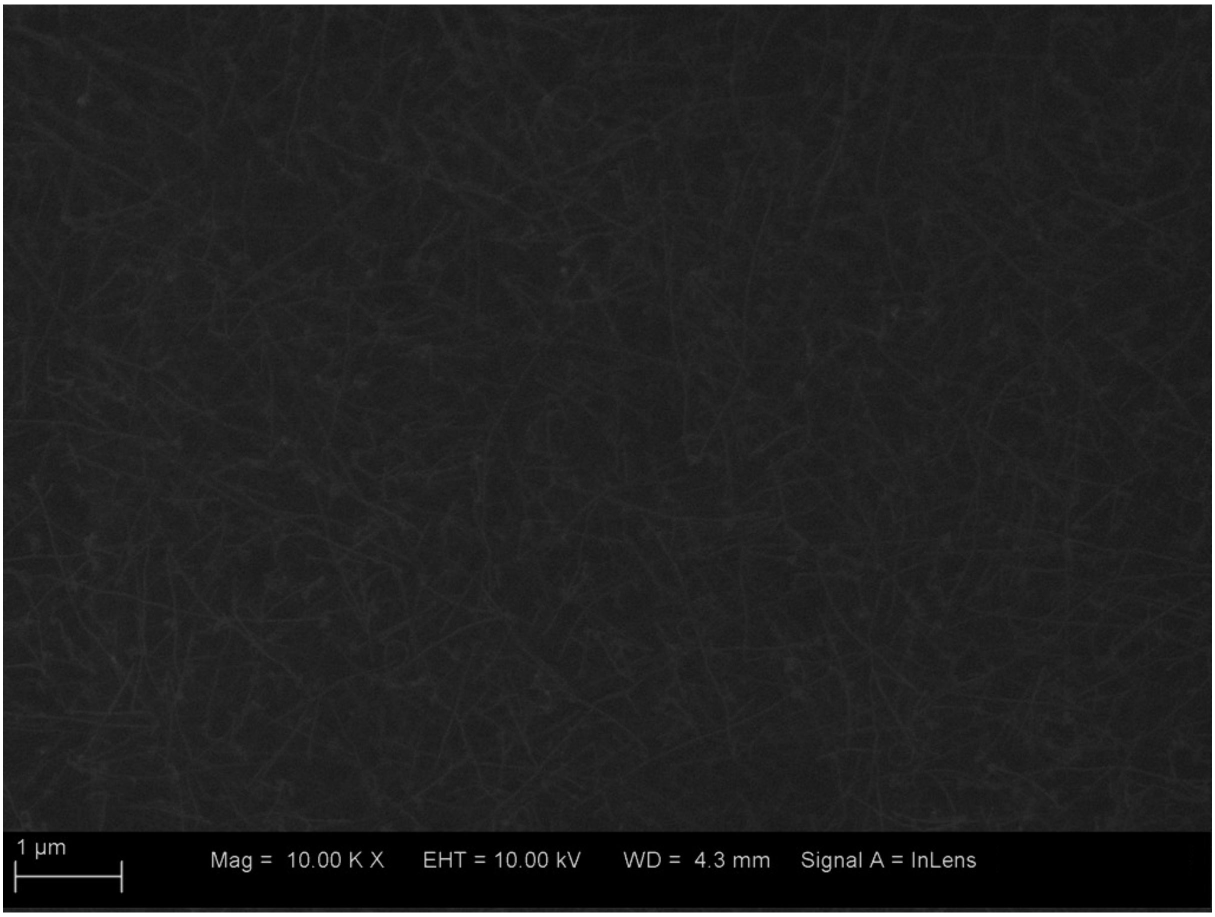

Figure S3: SEM picture of a composite with 1.61 wt% SWCNT.

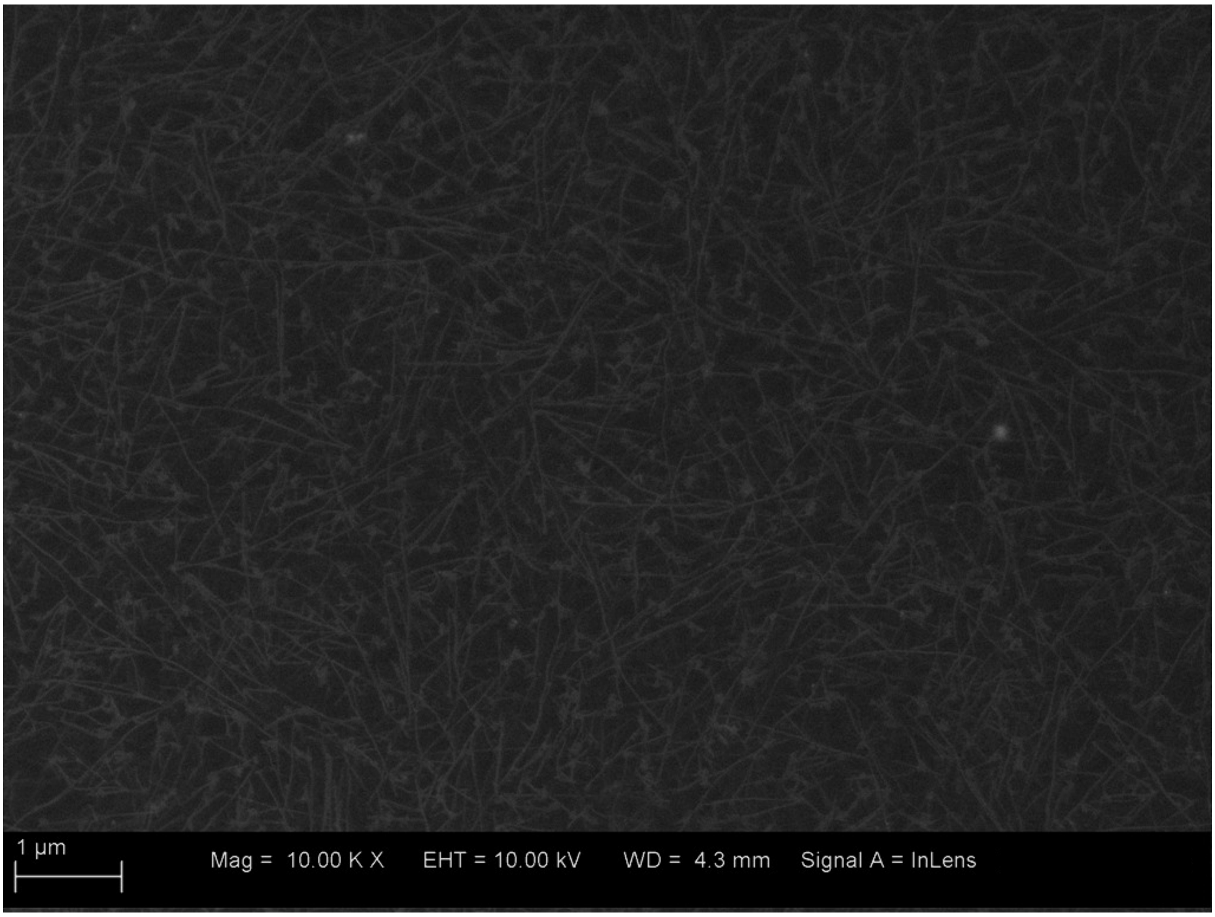

Figure S4: SEM picture of a composite with 3.12 wt% SWCNT.

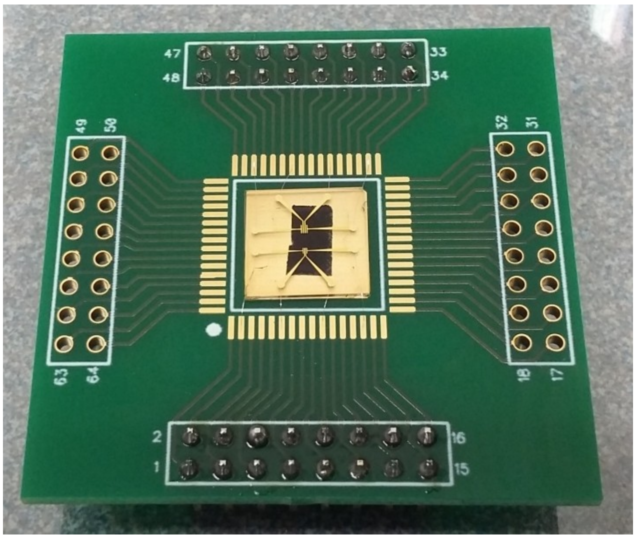

Figure S5: A typical device for electro-optical measurements. The BSi-substrate with a transparent 400 nm thick composite film on top is glued with carbon tape in the center of the PCB-board. Electrical connections between PCB-board and Au-electrodes on the sample are made by Al-wire bonding.

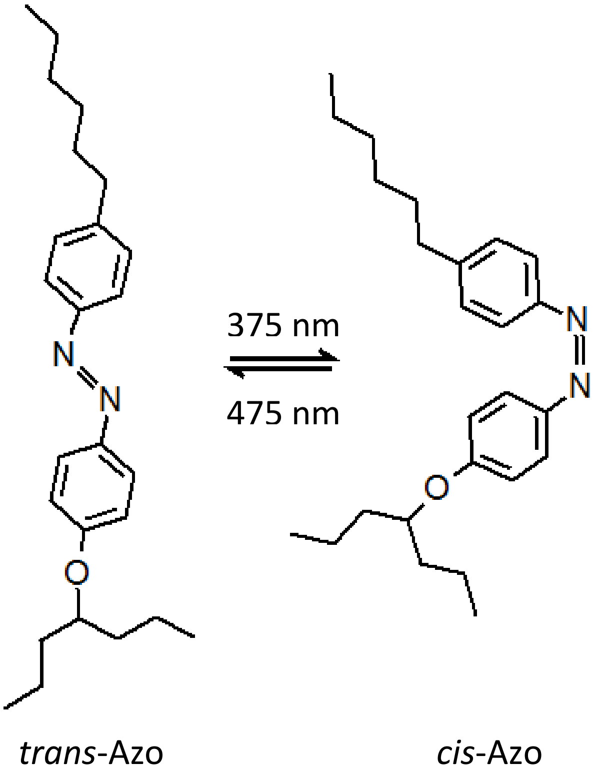

Figure S6: Azobenzene derivative used in this work. Photo-switching between the two isomer states was achieved by illumination with light of wavelengths of 375 nm and 475 nm, respectively. We note the reduction in distance between 4 and 4′ positions from 0.99 nm in the trans state to 0.55 nm in the cis state.[1]

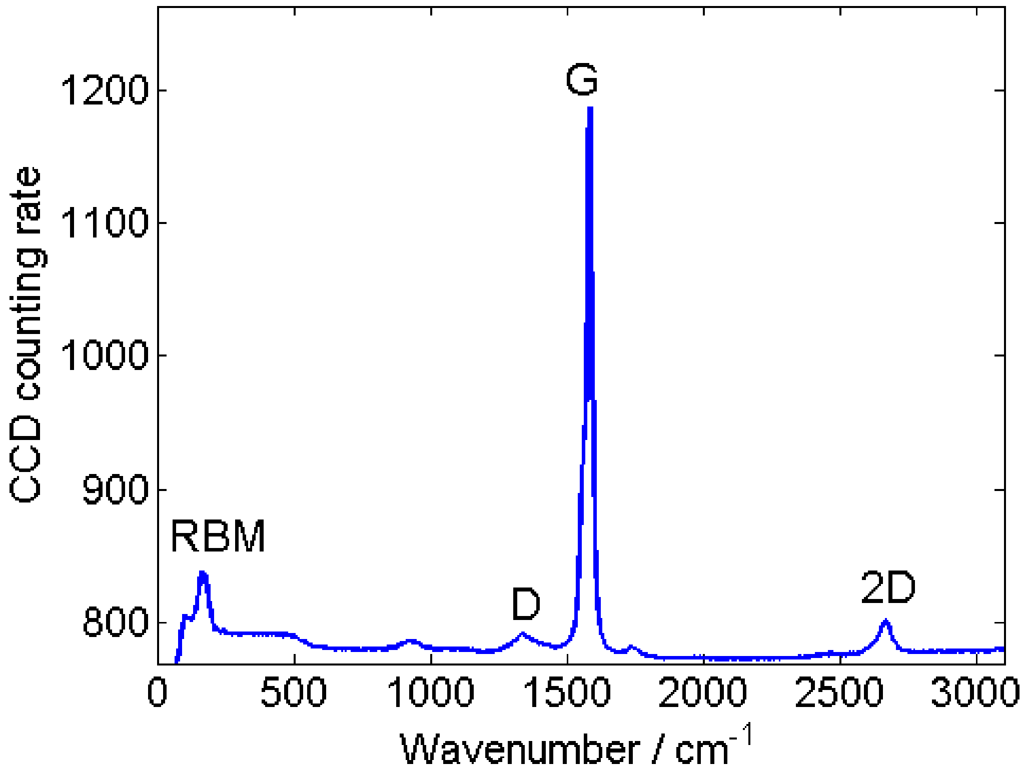

Figure S7: Acquired Raman spectra for the used SWCNT. The position of the Radial-Breathing-Mode (RBM) was found at 156.2 cm^{-1} with 30 cm^{-1} FWHM. The diameter was determined to d = 1.59 ± 0.16 nm according to [2]. The I(D)/I(G)-ratio was determined to 0.091.

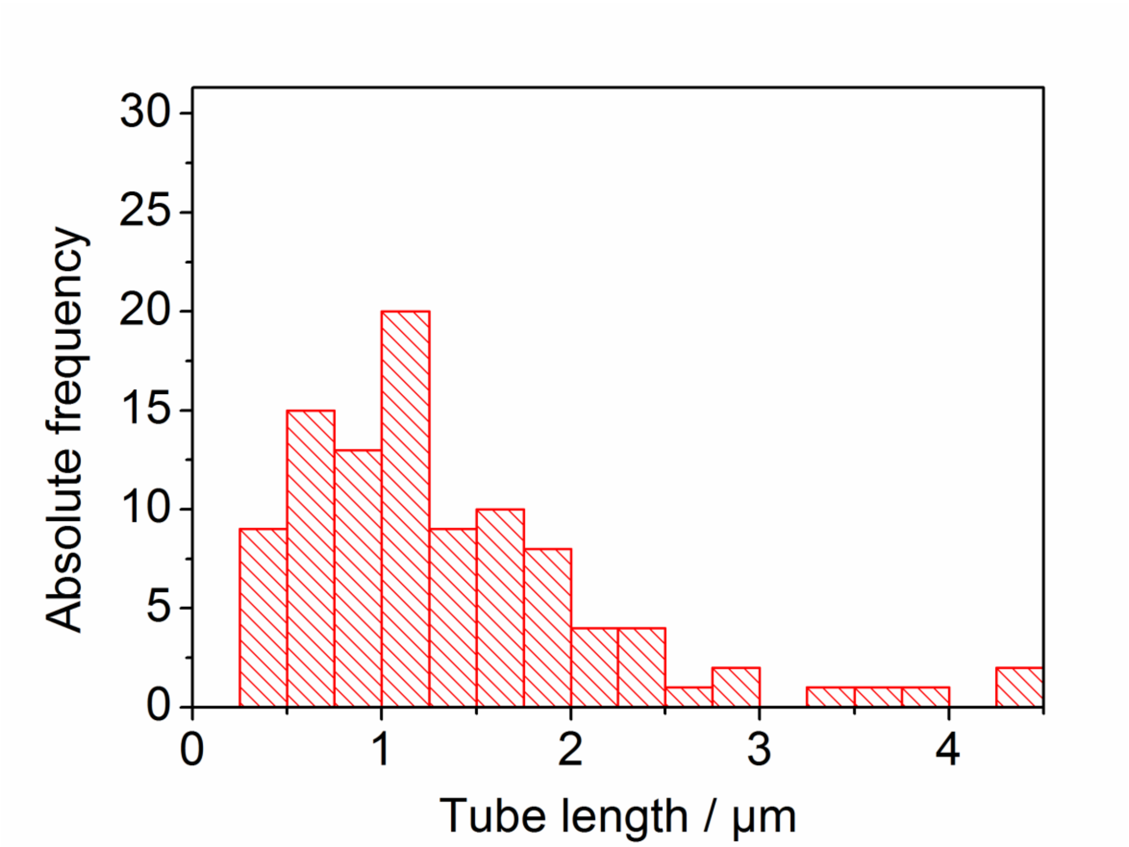

Figure S8: Length distribution of N=100 SWCNT's used in this work. The arithmetic average length was determined to l =1.35 µm.

1. Zhao, Y. & Ikeda, T. *SMART LIGHT- RESPONSIVE MATERIALS*. (John Wiley & Sons, Inc., Haboken, New Jersey, 2009).

2. Dresselhaus, M. S., Dresselhaus, G., Saito, R. & Jorio, A. Raman spectroscopy of carbon nanotubes. *Phys. Rep.* **409,** 47–99 (2005).
